# Cystatin C‐based eGFR better predicts renal vancomycin clearance than creatinine‐based eGFR in patients with allogeneic stem cell transplantation

**DOI:** 10.1002/cpt.70125

**Published:** 2025-11-12

**Authors:** Eva‐Maria A. Wansing, Sebastian G. Wicha, Peter Bannas, Alexander Heitkamp, Adrin Dadkhah, Nicolaus M. Kröger, Isabel Molwitz, Claudia Langebrake

**Affiliations:** ^1^ Hospital Pharmacy, University Medical Center Hamburg‐Eppendorf Hamburg Germany; ^2^ Department of Clinical Pharmacy Institute of Pharmacy, University of Hamburg Hamburg Germany; ^3^ Department of Diagnostic and Interventional Radiology and Nuclear Medicine University Medical Center Hamburg‐Eppendorf Hamburg Germany; ^4^ Department of Diagnostic and Interventional Neuroradiology University Medical Center Hamburg‐Eppendorf Hamburg Germany; ^5^ Department of Stem Cell Transplantation University Medical Center Hamburg‐Eppendorf Hamburg Germany

## Abstract

Knowledge of the glomerular filtration rate (GFR) is mandatory when dosing renally eliminated drugs such as vancomycin. In clinical practice, different biomarkers and various equations are used to estimate GFR (eGFR), resulting in varying estimates. These variations may be explained by nonrenal factors, such as muscle status or glucocorticoid administration. This study aimed to evaluate the performance of different eGFR equations in terms of accuracy and precision compared to renal vancomycin clearance, including subgroup analyses for nonrenal confounders. We retrospectively analyzed data from 121 adult allogeneic hematopoietic stem cell transplant (allo‐HSCT) patients. All patients received vancomycin treatment including trough concentration therapeutic drug monitoring. The eGFR was calculated using eight equations and compared to the renal vancomycin clearance that was calculated using a pharmacokinetic model and served as the reference. Individual muscle status was determined by computed tomography scans. Median renal vancomycin clearance was 49 mL/minute/1.73 m^2^ (range 24–96). All eight eGFR equations overestimated renal vancomycin clearance. The six (partially) creatinine‐based equations were significantly less accurate (bias: 24.0–62.8 mL/minute/1.73 m^2^) than both cystatin C‐based equations (bias: 6.3–9.5 mL/minute/1.73 m^2^). This decreased accuracy for creatinine‐based eGFR was more pronounced in patients with reduced muscle status or glucocorticoid medication. All CKD‐EPI equations and the Hoek equation were more precise with an IQR of the difference to renal vancomycin clearance ≤22.5 mL/minute/1.73 m^2^ compared to ≥35.5 mL/minute/1.73 m^2^ (Cockcroft‐Gault, MDRD). In conclusion, cystatin C‐based eGFR equations are preferable to creatinine‐based approaches for vancomycin dosing in allo‐HSCT patients.

**Study Highlights**

**WHAT IS THE CURRENT KNOWLEDGE ON THE TOPIC?**

The results of GFR estimation vary depending on the estimation equation used and the underlying biomarker. In cancer patients, the discrepancy between cystatin C‐ and creatinine‐based eGFR is greater than 30% for around half of patients, and this discrepancy is associated with CT‐defined sarcopenia. Previous studies suggest that estimation equations combining serum creatinine and cystatin C provide a more accurate prediction of eGFR in pediatric HSCT patients. Additionally, cystatin C‐based eGFR was found to be a more accurate and precise predictor of vancomycin clearance than creatinine‐based eGFR in other patient populations.

**WHAT QUESTION DID THIS STUDY ADDRESS?**

This study aimed to evaluate the performance of creatinine‐ and cystatin C‐based eGFR estimation equations compared to the renal vancomycin clearance in patients following allo‐HSCT, including subgroup analyses for concomitant glucocorticoid medication and individual muscle status.

**WHAT DOES THIS STUDY ADD TO OUR KNOWLEDGE?**

Cystatin C‐based eGFR estimation equations are preferable to creatinine‐based approaches for the dosing of renally excreted drugs such as vancomycin in patients undergoing allo‐HSCT. This is even more important in patients with compromised muscle status or concomitant glucocorticoid medication.

**HOW MIGHT THIS CHANGE CLINICAL PHARMACOLOGY OR TRANSLATIONAL SCIENCE?**

These results significantly improve routine patient care by enabling accurate dosing of drugs with a narrow therapeutic range, such as vancomycin. This prevents both overdosing, which can lead to side effects, and underdosing, which can result in treatment failure.

Knowledge of individual renal function is mandatory when dosing (predominantly) renally eliminated drugs, especially those with a narrow therapeutic range, such as vancomycin. The loading dose must be adjusted for individual body weight, whereas the initial maintenance dose is administered according to renal function until the first therapeutic drug monitoring (TDM) measurements are available.^1^, ^2^, ^3^

The renal function is usually expressed as glomerular filtration rate (GFR). The gold standard for determining GFR is the measured GFR (mGFR), calculated from plasma and urine data using the inulin method.^4^, ^5^ An alternative is the creatinine clearance, where creatinine, an endogenous marker, is measured in urine and plasma. However, these methods are time‐consuming and expensive. Alternatively, the GFR can be estimated (eGFR) by different equations based on serum concentrations of the biomarkers creatinine, cystatin C, or both.^6^, ^7^, ^8^, ^9^, ^10^

Creatinine is a product of muscle catabolism. Its serum concentration therefore depends on diet, sex, age, and muscle mass.^11^ However, muscle mass is often reduced after hematopoietic stem cell transplantation (HSCT).^12^ Individual muscle status, and therefore its potential impact on serum creatinine, is difficult to assess in routine clinical practice, as it is not well reflected by body weight or the body mass index.^13^, ^14^, ^15^ Therefore, CT or MRI is the established standard for determining muscle status, but this is not yet part of routine clinical practice. Both allow the determination of the Skeletal Muscle Index (SMI) as a determinant of muscle mass and muscle density as a determinant of myosteatosis and therefore a parameter of muscle quality.^16^, ^17^, ^18^ In contrast, cystatin C is produced in all nucleated cells and is presumably less dependent on muscle mass.^19^ However, it has been described to be influenced by nonrenal factors such as the concomitant administration of glucocorticoids.^20^, ^21^, ^22^, ^23^

Consequently, the results of GFR estimation differ depending on the estimation equation used. A study of cancer patients found that around half of the patients had a discrepancy of over 30% between cystatin C‐ and creatinine‐based eGFR, and that this discrepancy was associated with CT‐defined sarcopenia.^24^ Previous studies suggest a more accurate prediction of eGFR in pediatric HSCT patients when estimation equations combining serum creatinine and cystatin C are used.^25^, ^26^ In a prospective study in pediatric stem cell transplant patients, cystatin C or combined (cystatin C‐ and creatinine‐based) eGFR showed a better correlation with the clearance of the renally eliminated drug cefepime, than the creatinine‐based eGFR.^27^

However, there is no evidence and therefore no recommendation as to which of the commonly used eGFR estimation equations would be most accurate for vancomycin dosing in adult allo‐HSCT patients.

Therefore, the aim of this study was to evaluate the performance of creatinine‐ and cystatin C‐based eGFR estimation equations compared to the renal vancomycin clearance in patients following allo‐HSCT, including subgroup analyses for concomitant glucocorticoid medication and the individual muscle status. In this comparison, renal vancomycin clearance is not intended to serve as a direct substitute for mGFR, but rather to identify an eGFR estimation method that can be used to accurately determine the initial maintenance dose of vancomycin.

## MATERIALS AND METHODS

### Study design and population

This retrospective, observational study analyzed data from 121 patients. Inclusion criteria were adult patients with allo‐HSCT, an inpatient stay of at least 14 days at the Department of Stem Cell Transplantation at the University Medical Center Hamburg‐Eppendorf between January and December 2021, vancomycin treatment including documented trough concentrations from routine TDM within 7 days before or after a documented serum cystatin C and serum creatinine concentration, and if available a thoracic CT (TCT) scan from routine clinical care. Patients on dialysis during vancomycin treatment were excluded. TCT scans had to be within 14 days before or after a documented vancomycin trough concentration, as the individual muscle status derived from these scans was considered a potential factor influencing eGFR. Where available, up to three vancomycin trough concentrations per patient were included, with preference given to concentrations closest to the date of the CT scan.

We collected vancomycin trough concentrations, time and dose of vancomycin administration, age, sex, weight, height, serum creatinine, serum cystatin C, and underlying hematological disease from the electronic patient record. We also recorded the presence of concomitant systemic glucocorticoid medication (dexamethasone, hydrocortisone, methylprednisolone, or prednisolone administration for at least 48 hours within the last week before the vancomycin trough concentration, as it is assumed that the effect is delayed and persists until after the last glucocorticoid dose^22^, ^23^).

Vancomycin concentrations were quantified in‐house by a particle‐enhanced turbidimetric inhibition immunoassay (Atellica® CH Analyzer, Siemens Healthineers, Forchheim, Germany) with a detection range of 3–50 mg/l, at the Institute of Clinical Chemistry and Laboratory Medicine at the University Medical Center Hamburg‐Eppendorf.

This retrospective observational study received an ethics waiver (Ethics Committee of the Hamburg Medical Association (Ärztekammer), 2022‐300,185‐WF). It was conducted in compliance with the latest Declaration of Helsinki.

### Determination of SMI and muscle density

Conventionally, the SMI and muscle density are measured on axial slices at the height of the third lumbar vertebra.^28^ However, allo‐HSCT patients mostly receive TCT scans to search for pulmonary infection under immunosuppression. As TCT scans do not include the third lumbar vertebra, measurements were conducted on the mid‐height of the twelfth thoracic vertebrae. Good correlation between both heights has been demonstrated before.^29^ Axial CT slices were extracted from the picture archiving and communicating system. Measurements were conducted in the open‐source software ImageJ (National Institutes of Health, Laboratory for Optical and Computational Instrumentation, Wisconsin, USA). For the SMI, three regions of interest (ROIs) were defined along the outer thoracic muscle perimeter (ROI 1), the inner muscle perimeter (ROI 2), and around the circumference of the twelfth vertebrae (ROI 3). Fatty voxels were excluded by the muscle‐specific threshold of −29 to +150 Hounsfield units (HU) according to Gomez‐Perez et al.^30^ By subtraction of ROI 2 and ROI 3 from ROI 1 the muscle area was derived, which divided by body square height results in the SMI. The muscle density was derived as the mean HU value from the same muscle area after application of the muscle‐specific threshold.

Most of the patients in this study were below frequently used sarcopenia cutoff values (females: SMI < 30.6 cm^2^/m^2^, males <42.6 cm^2^/m^2^).^31^ In order to be able to differentiate eGFR estimates between patients with severely reduced and moderately reduced muscle status, patients were classified as below or above the sex‐specific median SMI (females: 25.6 cm^2^/m^2^, males: 31.4 cm^2^/m^2^) and muscle density value (females: 32 HU, males: 35 HU) of this cohort.

### eGFR calculation and determination of renal vancomycin clearance

We compared the renal vancomycin clearance as our reference clearance to the eGFR derived from eight different equations, as follows:

1. Cockcroft‐Gault^9^ based on serum creatinine (CG),
2. Cockcroft‐Gault^9^ based on serum creatinine and indexed to a body surface area (BSA) of 1.73 m^2^ (CG_indexed),
3. Modification of Diet in Renal Disease based on serum creatinine^8^ (MDRD),
4. Chronic Kidney Disease Epidemiology Collaboration (CKD‐EPI) based on serum creatinine^7^ (CKD‐EPI_SCR),
5. CKD‐EPI based on serum creatinine without race as a covariate^10^ (CKD‐EPI_NR),
6. CKD‐EPI based on serum creatinine and cystatin C^7^ (CKD‐EPI_combined),
7. CKD‐EPI based on serum cystatin C^7^ (CKD‐EPI_CYSC), and
8. Hoek equation based on serum cystatin C^6^ (HOEK).

The Cockcroft‐Gault equation was used in the original form and indexed to a BSA of 1.73 m^2^, for better comparability with the other equations, where the results are given in mL/minute/1.73 m^2^. For obese patients, adjusted ideal body weight (AIBW) was calculated using the following equation^32^ and was then used in both CG equations:

$$\text{AIBW}=\left(\text{total body weight}–\text{ideal body weight}\right)\times 0.4+\text{ideal body weight}$$

In the following, the term eGFR also refers to the Cockcroft‐Gault creatinine clearance.

To calculate the renal vancomycin clearance, we first calculated the plasma vancomycin clearance using the population pharmacokinetic model as published by Okada et al.^33^ that was recoded in NONMEM® (Version 7.5; Icon Development Solutions, Ellicot City, MD, USA) and applied it to the observed trough concentration data. This model demonstrated a good predictive performance in a model evaluation study that was conducted in this study cohort.^34^ Creatinine clearance was removed from the model as a covariate on plasma vancomycin clearance to avoid the introduction of bias on the estimation. The model showed sufficiently good predictive performance even without this covariate, with a median prediction error of −7.6% and a median absolute prediction error of 9.0%. According to the Summary of Product Characteristics, 83% of the plasma vancomycin clearance is eliminated via the kidneys.^1^ Therefore, we multiplied the plasma vancomycin clearance by 0.83 to obtain the renal vancomycin clearance. This renal clearance was then converted from L/hour to mL/minute and indexed to a BSA of 1.73 m^2^ to ensure comparability with the eGFR values expressed in mL/minute/1.73 m^2^.

Up to three observed vancomycin trough concentrations from routine TDM were recorded per patient. For each of these trough concentrations, the renal vancomycin clearance was calculated as described above.

### Statistics

#### Performance of different eGFR equations compared to the renal vancomycin clearance as a reference

All data manipulation and statistics were performed in R (version 4.3.2).^35^

The performance of each of the eight equations listed above was determined using the following metrics. Bias was calculated as the median difference between eGFR and renal vancomycin clearance as the reference. A bootstrap with *n* = 2,000 was used to calculate the 95% confidence interval (CI) of the bias. The IQR of the differences was used as a measure of precision, with a lower IQR indicating better precision. The p30 was calculated as the percentage of eGFR values that deviated less than or equal to 30% from the renal vancomycin clearance. In addition, renal vancomycin clearance and eGFR values were categorized into < 30, 30–45, 45–60, 60–90, and > 90 mL/minute according to the KDIGO eGFR categories.^36^ The percentage of cases in which the renal vancomycin clearance and eGFR categories matched was then calculated and used as a measure of agreement. Bias (absolute deviation), p30 (relative deviation), and the agreement were used as a measure of accuracy for each equation. To evaluate the clinical relevance of our results, we calculated the theoretical initial maintenance dose. We therefore compared the maintenance dose that would be administered according to our local eGFR‐based vancomycin dosing recommendations if creatinine or cystatin C were used, respectively. These local recommendations include five dosage steps, depending on the eGFR calculated using the CKD‐EPI equation and ranging from 3,000 mg/day for an eGFR of > 120 mL/minute/1.73 m^2^ to 500 mg/day for an eGFR of < 10 mL/minute/1.73 m^2^.

#### Impact of muscle status and concomitant glucocorticoid medication

The previously described metrics: bias, IQR, p30, and agreement were calculated for patients classified as below or above the sex‐specific median SMI and below or above the sex‐specific median muscle density. Between‐group differences in bias were assessed using Mann–Whitney *U* tests.

The same approach was used to assess differences between patients with and without concomitant glucocorticoid medication.

## RESULTS

### Study population

Baseline and longitudinal demographic and clinical data of the studied patient population are shown in **Table** **1**.

**Table 1:** Baseline and longitudinal (up to three data points per patient) demographic and clinical data of the patient population (*n* = 121)

	Total count (%) or median (range)
Baseline characteristics	
Male patients	73 (60.3)
Age (years)	58 (18–85)
Height (m)	1.76 (1.57–1.98)
Time difference from HSCT to the first vancomycin measurement (days)	7 (−8–6,615)
Vancomycin loading dose (mg/kg)	19.2 (7.8–27.2)
Underlying disease	
ALL	12 (9.9)
AML	37 (30.6)
Lymphoma	11 (9.1)
MDS	24 (19.8)
Myelofibrosis	19 (15.7)
Other	18 (14.9)
Longitudinal characteristics	
Weight (kg)	76.5 (43.2–137.7)
BMI (kg/m^2^)	25.2 (15.5–48.8)
SMI (cm^2^/m^2^)	28.6 (21.3–42.9)
Muscle density (HU)	35 (24–50)
Serum creatinine (mg/dL)	0.75 (0.36–1.87)
Serum cystatin C (mg/L)	1.26 (0.68–2.92)
Plasma vancomycin clearance (L/h)	3.94 (1.92–7.59)
Renal vancomycin clearance (L/h)	3.27 (1.60–6.30)
Renal vancomycin clearance (mL/minute/1.73 m^2^)	49 (24–96)
eGFR	
CG (mL/minute)	112 (39–267)
CG_ind (mL/minute/1.73 m^2^)	96 (28–251)
MDRD (mL/minute/1.73 m^2^)	96 (27–235)
CKD_SCR (mL/minute/1.73 m^2^)	98 (27–155)
CKD_NR (mL/minute/1.73 m^2^)	96 (27–144)
CKD_COMB (mL/minute/1.73 m^2^)	73 (21–139)
CKD_CYSC (mL/minute/1.73 m^2^)	56 (18–124)
HOEK (mL/minute/1.73 m^2^)	59 (23–114)

ALL, acute lymphocytic leukemia; AML, acute myelocytic leukemia; MDS, Myelodysplastic syndrome.

#### Performance of different eGFR equations compared to the renal vancomycin clearance as a reference

The median renal vancomycin clearance was 49 mL/minute/1.73 m^2^ (range 24–96 mL/minute/1.73 m^2^). All eight equations showed a significant bias in eGFR estimation with higher values as compared to the renal vancomycin clearance. However, there were also considerable differences between the equation results: The CKD‐EPI_CYSC equation had the lowest bias compared to the reference renal vancomycin clearance of 6.3 mL/minute/1.73 m^2^ (CI 4.6–10.2 mL/minute/1.73 m^2^). The other cystatin C‐based eGFR estimation equation (HOEK) also showed a low bias of 9.5 mL/minute/1.73 m^2^ (CI 7.9–11.2 mL/minute/1.73 m^2^).

The largest bias compared to the renal vancomycin clearance was found for the creatinine‐based CG equation with 62.8 mL/minute/1.73 m^2^ (CI 58.6–66.6 mL/minute/1.73 m^2^). Both equations based on cystatin C alone were significantly less biased than the creatinine‐based equations and the combined equation (*p <* 0.001). Although the combined equation was inferior to those equations based on cystatin C alone, it was significantly less biased than those based on creatinine alone (*p* < 0.001).

The p30 was below 10% for all creatinine‐based equations. It was higher for the CKD‐EPI_combined Eq. (26.1%) and above 50% for both cystatin C‐based equations (CKD‐EPI_CYSC: 55.4%, HOEK: 60.9%).

The agreement with the KDIGO criteria was below 10% for all creatinine‐based equations (**Figure** **S1**). It was higher for all equations based solely or partially on cystatin C (CKD‐EPI_combined: 16.8%, CKD‐EPI_CYSC: 40.3% and HOEK: 37.7%).

The lower IQR of all CKD‐EPI equations and the HOEK equation (each ≤22.5 mL/minute/1.73 m^2^) indicates a higher precision of these equations compared to the CG (IQR: 39.4 mL/minute/1.73 m^2^), CG_indexed (IQR: 37.6 mL/minute/1.73 m^2^), and MDRD (IQR: 35.5 mL/minute/1.73 m^2^) equations.

For 43% of patients, the initial maintenance dose based on CKD‐EPI_CYSC would have been equivalent to that based on CKD‐EPI_CREA. For 51% of patients, the dosage would have been reduced by one step and for 6% by two steps.

**Figure 1:**
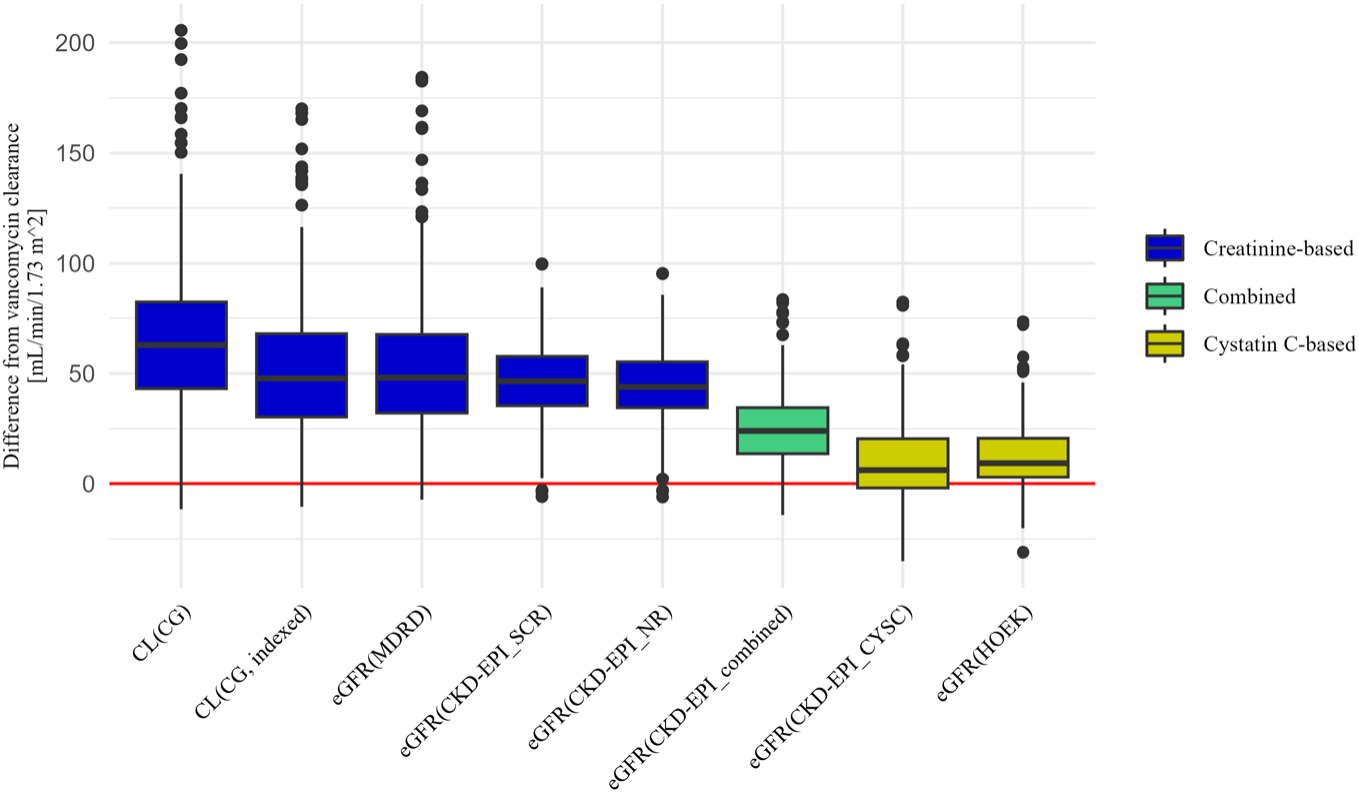
Differences between each eGFR and the renal vancomycin clearance to visualize bias and precision. Positive values indicate overprediction of renal vancomycin clearance, whereas negative values indicate underprediction. The red line indicates that there is no difference between the respective eGFR and renal vancomycin clearance.

**Figure 2:**
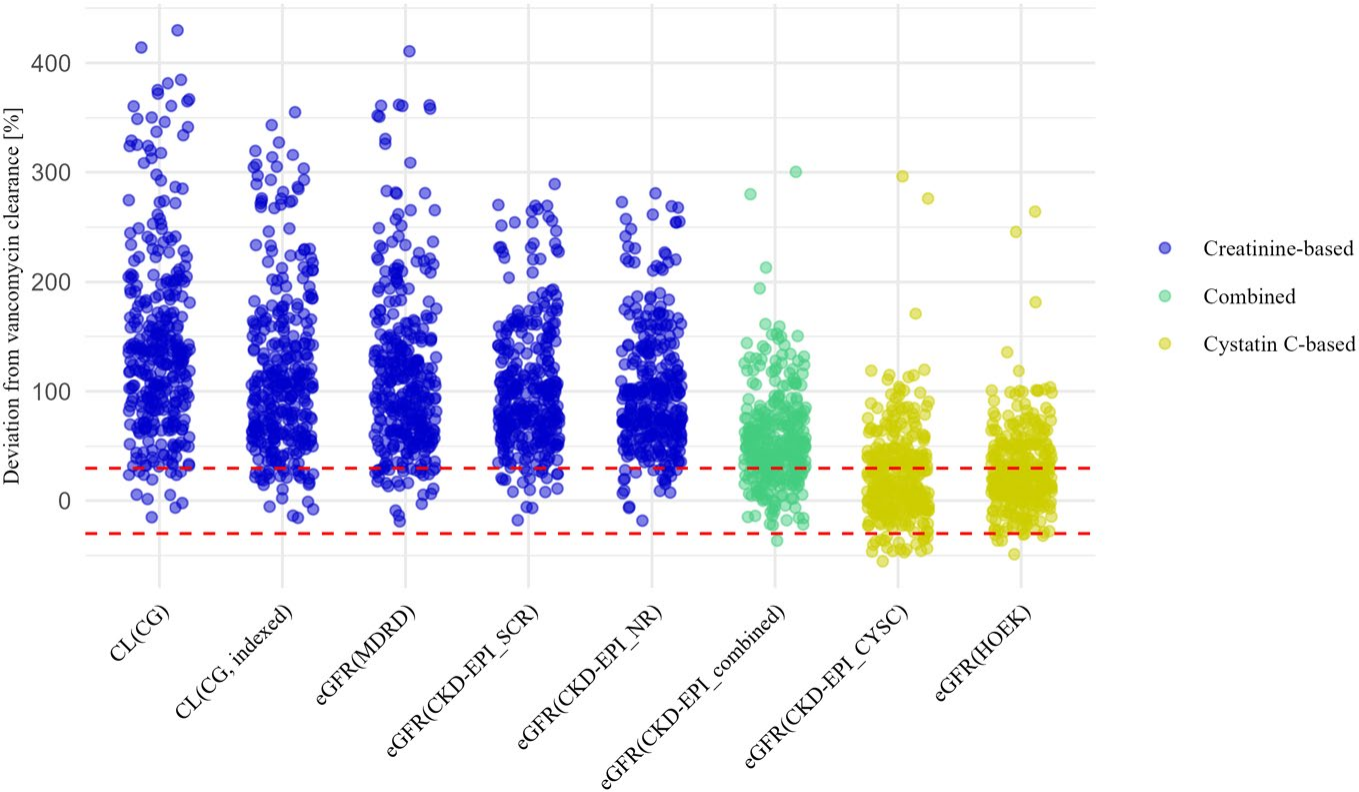
Scatter plots of the relative deviations between each eGFR and the renal vancomycin clearance. The values in the area between the red dashed lines represent the p30.

**Table** **2** and **Figures** **1** and **2** show the comparison between renal vancomycin clearance and the different eGFR equations.

**Table 2:** Evaluation of the different eGFR equations

Equation	Bias (mL/minute/1.73 m^2^)			IQR (mL/minute/1.73 m^2^)	p30 (%)	Agreement (%)
	Median	Lower CI	Upper CI			
CG	62.8	58.6	66.6	39.4	3.8	4.4
CG_indexed	47.8	44.9	52.6	37.6	8.7	5.5
MDRD	48.1	44.8	50.7	35.5	7.8	4.4
CKD‐EPI_SCR	46.5	43.1	49.0	22.3	5.5	3.2
CKD‐EPI_NR	43.9	41.5	46.6	20.7	6.1	4.1
CKD‐EPI_combined	24.0	21.1	25.5	20.8	26.1	16.8
CKD‐EPI_CYSC	6.3	4.6	10.2	22.5	55.4	40.3
HOEK	9.5	7.9	11.2	17.8	60.9	37.7

#### Impact of muscle status and concomitant glucocorticoid medication

There were 57 patients (47.1%) with vancomycin treatment and a TCT scan within 14 days before or after a documented vancomycin trough concentration. The sex‐specific median SMI was 25.6 cm^2^/m^2^ for females and 31.4 cm^2^/m^2^ for males and the sex‐specific median muscle density was 32 HU for females and 35 HU for males, respectively. The BMI (median, range) in this subgroup was significantly lower for patients below the sex‐specific median SMI (23.7 kg/m^2^, 19.9–29.8) than for patients above the sex‐specific median SMI (27.5 kg/m^2^, 19.6–48.5) (*p <* 0.001). It was significantly higher for patients below the sex‐specific median muscle density (27.2 kg/m^2^, 19.6–48.5) than for patients above the sex‐specific median muscle density (24.4 kg/m^2^, 19.9–34.0) (*p* = 0.0203). Our finding that creatinine‐based estimation equations were less accurate than those based on cystatin C was true for all patient groups, regardless of muscle status or glucocorticoid medication. This was particularly evident in patients with SMI or muscle density below the sex‐specific median, reflecting low muscle mass and myosteatosis. In these subgroups, the bias of all equations based on creatinine alone was higher than the bias of the same equation in the subgroups with SMI or muscle density above the sex‐specific median. These differences in bias between the corresponding subgroups were significant for the creatinine‐based equations MDRD (*p* = 0.0258), CKD‐EPI_SCR (*p* = 0.0332), and CKD‐EPI_NR (*p* = 0.0229) in patients with lower SMI and for CKD‐EPI_SCR (*p* = 0.0113) and CKD‐EPI_NR (*p* = 0.0060) in patients with lower muscle density.

The creatinine‐based equations showed a lower p30 and agreement in patients below the sex‐specific median SMI and muscle density than above the sex‐specific median. The only exception was the MDRD equation, where p30 was the same in the high and low muscle density groups. Estimation equations including serum cystatin C (CKD‐EPI_combined, CKD‐EPI_CYSC, and HOEK) did not show such a clear trend regarding the estimation performance between patients with higher or lower SMI or muscle density.

According to the aforementioned definition, concomitant glucocorticoid medication was present at 137 of the 345 measurement points at which eGFR and renal vancomycin clearance were determined in the 121 patients. In these cases, the creatinine‐based equations were significantly more biased than in cases without concomitant glucocorticoid medication (CG: *p* < 0.001, CG_indexed: *p* = 0.0010, MDRD: *p <* 0.001, CKD‐EPI_SCR: *p* = 0.0022, CKD‐EPI_NR: *p* = 0.0072). The p30 and the agreement were consistently higher in cases without concomitant glucocorticoid medication, regardless of the underlying biomarker.

**Figure 3:**
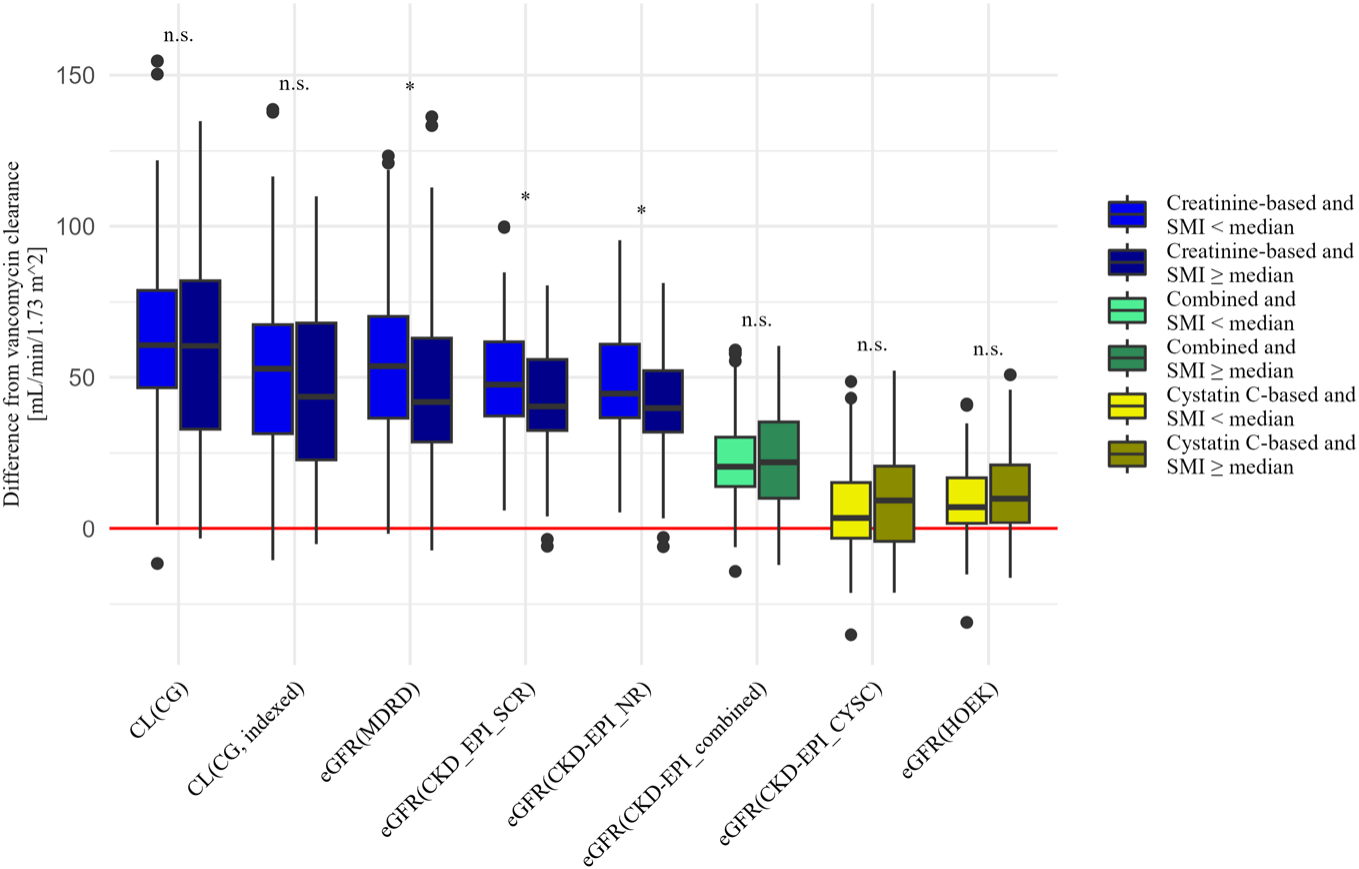
Comparison of patients with an SMI below or above the sex‐specific median, regarding the difference between each eGFR and the renal vancomycin clearance, to visualize bias and precision. The significance of the differences is labeled “n.s.” (not significant), “*” (*p <* 0.05), “**” (*p <* 0.01), and “***” (*p <* 0.001).

**Figure 4:**
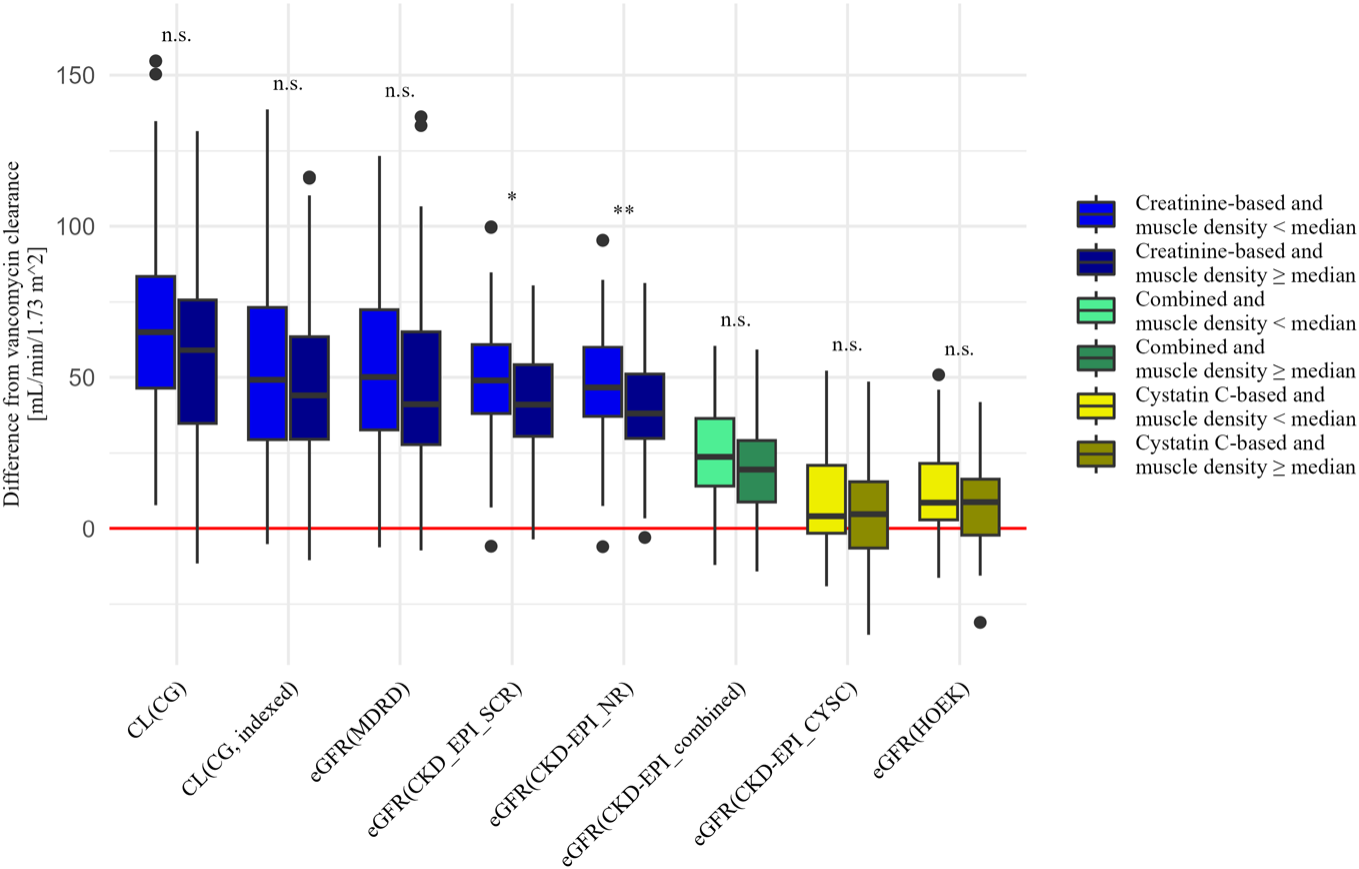
Comparison of patients with a muscle density below or above the sex‐specific median, regarding the difference between each eGFR and the renal vancomycin clearance, to visualize bias and precision. The significance of the differences is labeled “n.s.” (not significant), “*” (*p <* 0.05), “**” (*p <* 0.01), and “***” (*p <* 0.001).

**Figure 5:**
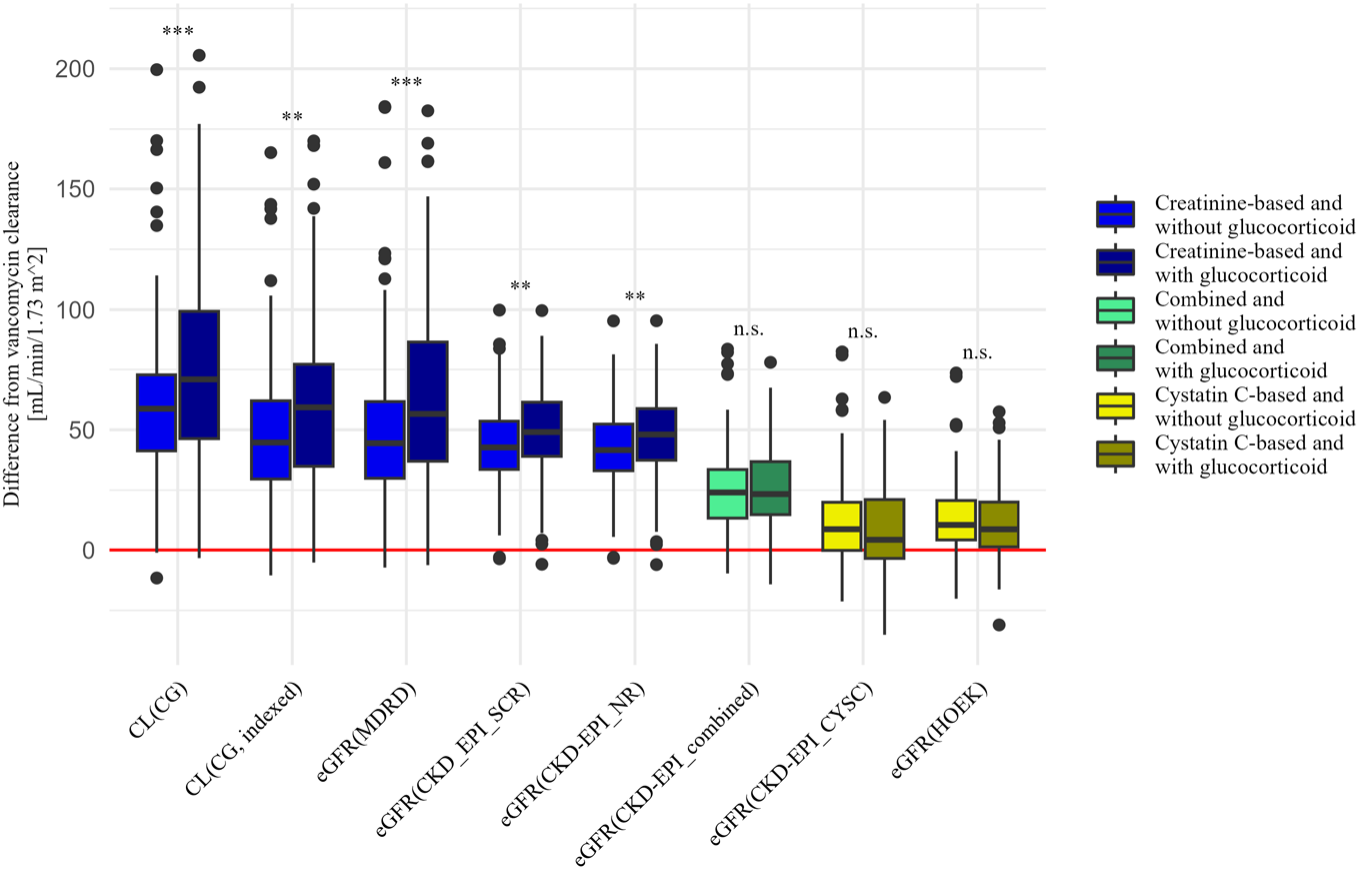
Comparison of patients with and without glucocorticoid medication, regarding the difference between each eGFR and the renal vancomycin clearance, to visualize bias and precision. The significance of the differences is labeled “n.s.” (not significant), “*” (*p <* 0.05), “**” (*p <* 0.01), and “***” (*p <* 0.001).

**Figures** **3**, **4**, **5** and **Table** **S1** show the comparison of renal vancomycin clearance and the different eGFR equations for patient groups below or above the sex‐specific median SMI (**Figure** **3**) or muscle density (**Figure** **4**) and with or without concomitant glucocorticoid medication (**Figure** **5**).

## DISCUSSION

This study evaluated the difference between the eGFR calculated using eight different equations and the renal vancomycin clearance as a reference, in adult allo‐HSCT patients. Subgroup analyses were performed according to muscle status or concomitant glucocorticoid medication. All eight GFR estimation equations overestimated the renal vancomycin clearance. However, the bias was smaller and still clinically acceptable for the cystatin C‐based equations at 6.3 mL/minute/1.73m^2^ (CKD‐EPI_CYSC) and 9.5 mL/minute/1.73m^2^ (HOEK). All eGFR equations based solely or partially on creatinine showed an unacceptably large bias. These findings were more pronounced in patients with reduced muscle status or concomitant glucocorticoid medication.

A systematic review and meta‐analysis concluded that cystatin C‐based eGFR was more accurate and precise than creatinine‐based methods at predicting vancomycin clearance in different populations.^37^ However, to our knowledge, such findings have not yet been published for adult allo‐HSCT patients. Previous data suggested that combined estimation equations (based on both creatinine and cystatin C) best reflect renal function in pediatric allo‐HSCT patients.^25^, ^26^ Furthermore, combined eGFR was reported to correlate better with measured creatinine clearance than either creatinine or cystatin C‐based eGFR alone in adult patients after allo‐HSCT.^38^ In contrast, in our patient cohort, we found that cystatin C‐based estimation equations predicted renal vancomycin clearance significantly better than those based on creatinine, while the combined eGFR equation was in between. To our knowledge, this is the first study to compare different eGFR estimation methods with renal vancomycin clearance in allo‐HSCT patients, taking into account potential confounders such as concomitant glucocorticoid medication and individual muscle status.

Regarding the impact of the muscle status, we found that creatinine‐based equations are more biased in allo‐HSCT patients with severely reduced muscle status, although these differences were not always significant. This is comprehensible, as creatinine is influenced by muscle mass, and highlights that cystatin C‐based equations are more accurate in predicting renal vancomycin clearance in allo‐HSCT patients, who often have reduced muscle status^12^. Frequent use of glucocorticoids, intensive pretreatment, malignancy, and reduced mobility are likely to contribute to this reduced muscle status. To our knowledge, the influence of muscle quality on creatinine‐based eGFR prediction is less well known than the influence of muscle mass. However, we have demonstrated that creatinine‐based equations are also influenced by muscle quality as indicated by the stronger bias in patients with low muscle density, which indicates myosteatosis. Therefore, image‐based body composition analyses are beneficial in identifying HSCT patients with severely reduced muscle mass. For these patients, cystatin C‐based eGFR estimation would be particularly useful.

Regarding concomitant glucocorticoid medication, all eGFR estimation methods performed better in terms of p30 and agreement in patients without glucocorticoids. In addition, all creatinine‐based equations were significantly less biased in patients without glucocorticoid medication. It has been previously described that glucocorticoid administration leads to an increased serum cystatin C and therefore decreased cystatin C‐based eGFR.^20^, ^21^, ^22^, ^23^ On the other hand, glucocorticoid‐induced muscle wasting could lead to a lower serum creatinine and therefore a higher creatinine‐based eGFR.^39^, ^40^ In conclusion, both biomarkers are affected by glucocorticoids, but in opposite ways. This may explain our finding that p30 and agreement were always better in patients without concomitant glucocorticoid medication and that all creatinine‐based equations were significantly less biased in this group. Although the differences were not significant, the cystatin C‐based equations demonstrated less bias in patients on glucocorticoids. This is consistent with the previously mentioned facts that (i) all equations overestimate renal vancomycin clearance, and (ii) glucocorticoids result in a lower cystatin C‐based eGFR, leading to less overestimation and reduced bias.

Our study had some limitations. First, we compared the different eGFR against renal vancomycin clearance as a reference instead of an mGFR. Moreover, renal vancomycin clearance was used because the aim of this study was to evaluate eGFR estimation methods for dosing of a renally eliminated drug under standard clinical conditions. This methodology may not be applicable to other research questions. In addition, we assumed a renally eliminated fraction of 83% for vancomycin, as stated in the Summary of Product Characteristics.^1^ Some studies report different data for the renal elimination fraction. Therefore, 83% may not be appropriate for all patient populations, and this fraction may also differ in patients with renal impairment.^41^, ^42^ However, assuming a lower renally eliminated fraction would result in greater overestimation by all tested equations, while cystatin C‐based equations remain less biased than creatinine‐based equations. Therefore, this assumption would not affect the main finding of this study. Due to the retrospective study design, the time data of vancomycin administration and sampling may not be documented as accurately as in prospective studies. However, it can be assumed that the estimation of the vancomycin clearance is fairly accurate through the use of a model‐informed approach.^43^ Reduced muscle status was defined based on the sex‐specific median SMI and median muscle density of this cohort, as most of the patients would have been below the commonly employed cutoff values for sarcopenia reported in the literature.^31^ Thus, the whole population studied tends to be sarcopenic, and the patients below the sex‐specific median SMI or muscle density have a severely reduced muscle status.

In conclusion, cystatin C‐based eGFR estimation equations are preferable to creatinine‐based approaches for the dosing of renally excreted drugs such as vancomycin in patients undergoing allo‐HSCT. This is even more important in patients with compromised muscle status or concomitant glucocorticoid medication. It is important to note that our findings may not apply to other patient populations and other drugs.

## FUNDING

No funding was received for this work.

## CONFLICT OF INTEREST

The authors declared no conflict of interest.

## AUTHOR CONTRIBUTIONS

E.A.W. and I.M. wrote the manuscript; E.A.W., S.G.W., P.B., N.M.K, I.M., and C.L. designed the research; E.A.W., A.H., A.D., I.M., and C.L. performed the research; E.A.W. analyzed the data.

## Supporting information


Figure S1 and Table S1.
