# Supplementary material for: Cystatin C‐based eGFR better predicts renal vancomycin clearance than creatinine‐based eGFR in patients with allogeneic stem cell transplantation
Source: Clin Pharmacol Ther. 2025 Nov 12;119(3):669–77. doi: 10.1002/cpt.70125 (PMC12882760; doi:10.1002/cpt.70125)
Supplement: Supplementary file 1 — Figure S1 and Table S1. [file CPT-119-669-s001.docx]

**Supplementary Material**

**
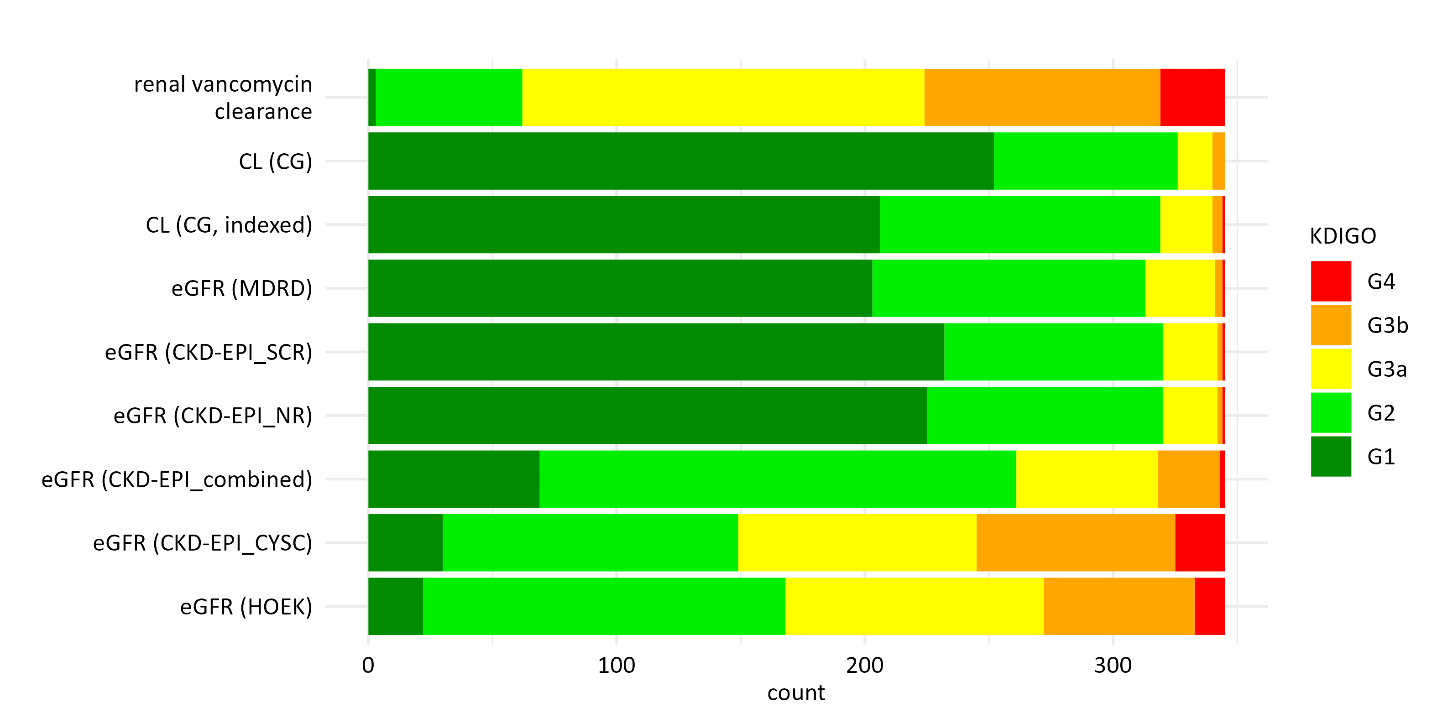
**

**Figure S1.** Bar chart showing the categorization of each eGFR alongside the renal vancomycin clearance, according to the KDIGO eGFR categories.

**Table S1.** Evaluation of the different eGFR equations for patients grouped by SMI below or above the sex-specific-median, muscle density below or above sex-specific-median and with or without concomitant glucocorticoid medication.

| **Equation** | **Patient group** | **Bias**  **[mL/min/1.73 m^2^]** | | | **IQR**  **[mL/min/1.73 m^2^]** | **P30 [%]** | **Agreement [%]** |
| --- | --- | --- | --- | --- | --- | --- | --- |
|  |  | **Median** | **Lower CI** | **Upper CI** |  |  |  |
| CG | SMI below median | 60.7 | 58.4 | 67.7 | 32.3 | 1.2 | 0.9 |
| CG | SMI above median | 60.4 | 51.2 | 69.8 | 49.1 | 1.5 | 2.3 |
| CG | Muscle density below median | 65.1 | 58.1 | 73.2 | 37.0 | 0.3 | 1.2 |
| CG | Muscle density above median | 59.0 | 51.0 | 64.4 | 40.9 | 2.3 | 2.0 |
| CG | Without glucocorticoid medication | 58.7 | 55.1 | 64.6 | 31.5 | 2.6 | 3.2 |
| CG | With glucocorticoid medication | 70.9 | 60.4 | 78.1 | 52.9 | 1.2 | 1.2 |
| CG_indexed | SMI below median | 52.8 | 44.6 | 61.4 | 36.1 | 2.0 | 1.5 |
| CG_indexed | SMI above median | 43.6 | 37.6 | 49.2 | 45.3 | 2.9 | 2.3 |
| CG_indexed | Muscle density below median | 49.2 | 42.1 | 61.2 | 43.8 | 1.2 | 1.2 |
| CG_indexed | Muscle density above median | 44.0 | 36.8 | 54.5 | 34.0 | 3.8 | 2.6 |
| CG_indexed | Without glucocorticoid medication | 44.7 | 42.0 | 48.0 | 32.4 | 4.9 | 4.1 |
| CG_indexed | With glucocorticoid medication | 59.3 | 48.4 | 63.6 | 42.3 | 3.8 | 1.5 |
| MDRD | SMI below median | 53.7 | 42.3 | 60.8 | 33.7 | 1.2 | 0.3 |
| MDRD | SMI above median | 41.9 | 32.8 | 48.7 | 34.4 | 2.9 | 2.0 |
| MDRD | Muscle density below median | 50.1 | 43.9 | 58.9 | 39.8 | 2.0 | 0.9 |
| MDRD | Muscle density above median | 41.1 | 34.1 | 53.8 | 37.4 | 2.0 | 1.5 |
| MDRD | Without glucocorticoid medication | 44.5 | 40.6 | 47.8 | 31.8 | 5.2 | 2.9 |
| MDRD | With glucocorticoid medication | 56.6 | 50.3 | 62.6 | 49.6 | 2.6 | 1.5 |
| CKD-EPI_SCR | SMI below median | 47.6 | 41.6 | 50.9 | 24.6 | 1.5 | 0.6 |
| CKD-EPI_SCR | SMI above median | 40.4 | 37.4 | 48.4 | 23.4 | 2.3 | 1.7 |
| CKD-EPI_SCR | Muscle density below median | 49.0 | 45.0 | 54.0 | 22.9 | 1.2 | 0.9 |
| CKD-EPI_SCR | Muscle density above median | 41.0 | 36.7 | 45.5 | 23.6 | 2.6 | 1.5 |
| CKD-EPI_SCR | Without glucocorticoid medication | 42.7 | 40.2 | 47.7 | 20.0 | 3.2 | 2.0 |
| CKD-EPI_SCR | With glucocorticoid medication | 49.1 | 44.8 | 55.6 | 22.4 | 2.3 | 1.2 |
| CKD-EPI_NR | SMI below median | 44.6 | 39.9 | 48.9 | 24.4 | 2.0 | 1.5 |
| CKD-EPI_NR | SMI above median | 39.8 | 36.1 | 43.9 | 20.2 | 2.3 | 1.7 |
| CKD-EPI_NR | Muscle density below median | 46.7 | 41.5 | 51.0 | 22.9 | 1.2 | 0.9 |
| CKD-EPI_NR | Muscle density above median | 38.1 | 36.0 | 42.5 | 21.2 | 3.2 | 2.3 |
| CKD-EPI_NR | Without glucocorticoid medication | 41.6 | 39.5 | 44.3 | 19.3 | 3.8 | 2.9 |
| CKD-EPI_NR | With glucocorticoid medication | 48.1 | 43.7 | 51.8 | 21.4 | 2.3 | 1.2 |
| CKD-EPI_combined | SMI below median | 20.5 | 18.0 | 24.1 | 16.3 | 7.8 | 3.2 |
| CKD-EPI_combined | SMI above median | 22.0 | 17.6 | 27.4 | 25.2 | 7.0 | 5.8 |
| CKD-EPI_combined | Muscle density below median | 23.8 | 19.2 | 28.2 | 22.3 | 5.2 | 3.8 |
| CKD-EPI_combined | Muscle density above median | 19.6 | 16.6 | 24.1 | 20.3 | 9.6 | 5.2 |
| CKD-EPI_combined | Without glucocorticoid medication | 24.1 | 21.2 | 26.6 | 20.1 | 16.5 | 9.9 |
| CKD-EPI_combined | With glucocorticoid medication | 23.4 | 19.8 | 26.1 | 21.9 | 9.6 | 7.0 |
| CKD-EPI_CYSC | SMI below median | 3.6 | 2.4 | 5.5 | 18.5 | 15.1 | 9.0 |
| CKD-EPI_CYSC | SMI above median | 9.4 | 2.9 | 14.0 | 25.0 | 12.5 | 9.6 |
| CKD-EPI_CYSC | Muscle density below median | 4.2 | 2.4 | 14.0 | 22.5 | 13.9 | 11.6 |
| CKD-EPI_CYSC | Muscle density above median | 4.9 | 2.4 | 9.3 | 22.0 | 13.6 | 7.0 |
| CKD-EPI_CYSC | Without glucocorticoid medication | 8.6 | 5.0 | 11.7 | 20.1 | 34.5 | 25.5 |
| CKD-EPI_CYSC | With glucocorticoid medication | 4.2 | 1.4 | 11.2 | 24.6 | 20.9 | 14.8 |
| HOEK | SMI below median | 7.2 | 5.2 | 9.4 | 15.2 | 14.5 | 9.6 |
| HOEK | SMI above median | 10.0 | 7.5 | 16.3 | 19.1 | 14.2 | 7.8 |
| HOEK | Muscle density below median | 8.7 | 6.6 | 16.3 | 18.6 | 14.2 | 10.1 |
| HOEK | Muscle density above median | 8.8 | 5.1 | 11.4 | 18.6 | 14.5 | 7.3 |
| HOEK | Without glucocorticoid medication | 10.4 | 8.3 | 12.2 | 16.5 | 37.1 | 22.0 |
| HOEK | With glucocorticoid medication | 8.6 | 5.7 | 11.6 | 18.8 | 23.8 | 15.7 |
